# Reproducible Identification of *Staphylococcus aureus* Bacteremia Clinical Subphenotypes

**DOI:** 10.1093/cid/ciaf655

**Published:** 2025-11-27

**Authors:** Maaike C Swets, Zsuzsa Bakk, Annette C Westgeest, Felicia Ruffin, Rachel Korn, Jean-Francois Jabbour, Priscilla La, Luke Tysall, Simon Dewar, Rebecca K Sutherland, Mark G J de Boer, Geert H Groeneveld, David H Dockrell, Thomas W van der Vaart, Vance G Fowler, Clark D Russell, IDISA study team, Jan M Prins, Robin Soetekouw, Gitte van Twillert, Jan Veenstra, Bjorn L Herpers, Wouter Rozemeijer, Rogier R Jansen, Marc J M Bonten, Jan T M van der Meer

**Affiliations:** Department of Infectious Diseases, Leiden University Medical Center, Leiden University, Leiden, The Netherlands; Department of Internal Medicine, Haaglanden Medical Center, The Hague, The Netherlands; Department of Methodology and Statistics, Leiden University, Leiden, The Netherlands; Department of Infectious Diseases, Leiden University Medical Center, Leiden University, Leiden, The Netherlands; Division of Infectious Diseases and International Health, Department of Medicine, Duke University School of Medicine, Durham, North Carolina, USA; Division of Infectious Diseases and International Health, Department of Medicine, Duke University School of Medicine, Durham, North Carolina, USA; Division of Infectious Diseases and International Health, Department of Medicine, Duke University School of Medicine, Durham, North Carolina, USA; Division of Infectious Diseases and International Health, Department of Medicine, Duke University School of Medicine, Durham, North Carolina, USA; Medical Microbiology, Royal Infirmary of Edinburgh, Edinburgh, United Kingdom; Medical Microbiology, Royal Infirmary of Edinburgh, Edinburgh, United Kingdom; Clinical Infection Research Group, Western General Hospital, Edinburgh, United Kingdom; Clinical Infection Research Group, Western General Hospital, Edinburgh, United Kingdom; Department of Infectious Diseases, Leiden University Medical Center, Leiden University, Leiden, The Netherlands; Department of Infectious Diseases, Leiden University Medical Center, Leiden University, Leiden, The Netherlands; Department of Internal Medicine-Acute Internal Medicine, Leiden University Medical Center, Leiden, The Netherlands; Centre for Inflammation Research, Institute for Regeneration and Repair, The University of Edinburgh, Edinburgh, United Kingdom; Division of Infectious Diseases, Department of Internal Medicine, Amsterdam UMC, University of Amsterdam, Amsterdam, The Netherlands; Division of Infectious Diseases and International Health, Department of Medicine, Duke University School of Medicine, Durham, North Carolina, USA; Infectious Diseases, Duke Clinical Research Institute, Durham, North Carolina, USA; Centre for Inflammation Research, Institute for Regeneration and Repair, The University of Edinburgh, Edinburgh, United Kingdom

**Keywords:** *Staphylococcus aureus*, bacteremia, patient stratification, subphenotypes, MRSA

## Abstract

**Background:**

Clinical heterogeneity in *Staphylococcus aureus* bacteremia (SAB) complicates clinical management and research. We have previously identified 5 clinically distinct subphenotypes of SAB associated with differences in outcomes and response to adjunctive rifampicin. Here, we aimed to identify these subphenotypes in geographically diverse observational cohorts, including a higher prevalence of methicillin-resistant *S. aureus* (MRSA) bacteremia and the USA300 clone.

**Methods:**

We studied 3 cohorts of adults with SAB from observational studies: a UK retrospective study (Edinburgh cohort 2; n = 463); a Dutch prospective study (IDISA [Improved Diagnostic Strategies in *Staphylococcus aureus* bacteremia study]; n = 490); and a prospective US study (SABG-PCS [*Staphylococcus aureus* Bacteremia Group Prospective Cohort Study]; n = 755). Subphenotypes were identified from routinely available clinical data using latent class analysis.

**Results:**

Patients from the SABG-PCS cohort had greater multimorbidity and more MRSA bacteremia (40.2% [303 of 755]), including infection with the USA300 clone (14.7% [111 of 755]). Five distinct subphenotypes were identified in each cohort: (1) older age and cardiometabolic multimorbidity; (2) nosocomial acquisition and intravenous catheter portal of entry; (3) community acquisition and metastatic infection; (4) chronic kidney disease; and (5) younger age, injection drug use, and metastatic infection. Bacterial genotypes varied substantially between the Edinburgh 2 and SABG-PCS cohorts but did not differ between subphenotypes within each cohort. 90-day mortality was highest in subphenotype A, and persistent bacteremia in subphenotypes C and E.

**Conclusions:**

We have reproducibly identified 5 clinical subphenotypes of SAB in observational cohorts including diverse bacterial genetic lineages and a cohort with a high prevalence of MRSA and USA300 bacteremia. These robustly reproducible clinical subphenotypes provide a framework to rationalize the heterogeneity intrinsic to SAB.

## Teaser

We report the reproducible identification of 5 clinical subphenotypes in 3 observational cohorts of *Staphylococcus aureus* bacteremia (n = 1708) from the United Kingdom, the Netherlands, and the United States, including a cohort with a high prevalence of methicillin-resistant *S. aureus*.

*Staphylococcus aureus* bacteremia (SAB) is a common and complicated infection, globally accounting for the largest number of deaths associated with bacteremia [1, 2]. In addition to its mortality rate, SAB can be complicated by metastatic foci of infection, recurrence despite seemingly appropriate treatment, and associated morbidity [3, 4]. The heterogeneity intrinsic to SAB challenges the efficacy of universal treatment approaches, complicating the management of individual patients in addition to research efforts to improve treatment outcomes [5]. In our group’s previous work, we demonstrated that this heterogeneity can be rationalized using latent class analysis (LCA) to identify 5 subphenotypes of SAB representing clinically distinct and readily interpretable disease archetypes, formed without consideration of outcome data [6, 7].

Supporting the clinical relevance of this approach, these subphenotypes were associated with differences in mortality and microbiologic outcomes and differential treatment effects of adjunctive rifampicin in the ARREST trial [8]. These findings were based on 3 cohorts from the United Kingdom and Spain, 2 of which were from clinical trials (ARREST and SAFO [4, 8]), including only 64 of 1430 patients with methicillin-resistant *S. aureus* (MRSA) bacteremia.

The generalizability of these findings to “real-life” patient cohorts in general, and MRSA bacteremia specifically, remains unresolved. Epidemiologic differences such as the prevalence of MRSA and the hypervirulent USA300 clone differ significantly across regions and are known to influence outcomes [9–11]. To address this, we aimed to replicate the identification of these previously identified subphenotypes using 3 geographically diverse observational cohorts, including 1 from the United States with a higher prevalence of MRSA bacteremia and the USA300 clone.

## METHODS

### Patient Cohorts

Patients were included from 3 observational studies: a cohort from an ongoing UK (Scotland) retrospective study (Edinburgh cohort 2; n = 463) [3, 6]; the Dutch IDISA (Improved Diagnostic Strategies in *Staphylococcus aureus* bacteremia study) multicenter prospective study (IDISA cohort; n = 490) [12], and the US SABG-PCS (*Staphylococcus aureus* Bacteremia Group Prospective Cohort Study) prospective study (SABG-PCS cohort; n = 755) [10]. The Edinburgh 2 cohort included consecutive unique adults (aged ≥18 years) with monomicrobial SAB diagnosed between 28 August 2022 and 18 December 2024, approved by the South East Scotland Research Ethics Committee. No patients included in our previous analysis were included in this second cohort of patients. The IDISA cohort enrolled adults (aged ≥18 years) with SAB between 1 July 2017 to 30 September 2019, from 7 hospitals in the Randstad metropolitan region of the Netherlands, approved by the Medical Ethics Committee of the Academic Medical Centre Amsterdam. The cohort from SABG-PCS analyzed in this study included adults (aged ≥18 years) with monomicrobial SAB enrolled 1 January 2016 to 1 January 2020, at Duke University Medical Center in North Carolina, approved by the Duke Institutional Review Board.

### Variable Definitions

The Charlson Comorbidity Index (CCI) was used to define comorbid conditions, with adjustment for age. Vascular disease was defined as a history of peripheral vascular disease, myocardial infarction, or stroke. The route of acquisition of infection was classified based on the criteria outlined by Friedman et al [13]. The portal of entry was defined as the most probable entry point of *S. aureus* into the bloodstream. Metastatic infection was defined as the clinical, radiologic, or microbiologic identification of foci of infection remote from the portal of entry, thought to have arisen through hematogenous dissemination, and it included infective endocarditis.

In the SABG-PCS cohort, heart rate, temperature, and creatinine level were collected as ordinal variables (Supplementary Table 1). For the Edinburgh 2 and IDISA cohorts, they were continuous variables. In the IDISA cohort, recorded vital signs and laboratory measurements were the most extreme values within 24 hours of the index blood culture. In the Edinburgh 2 and SABG-PCS cohorts, vital signs and laboratory measurements were recorded closest to the time of the index blood culture. In the Edinburgh 2 cohort, *spa* typing and Panton-Valentine leukocidin (*pvl*) gene quantitative reverse-transcription polymerase chain reaction were performed by the Scottish Microbiology Reference Laboratory. In the SABG-PCS cohort, *spa* typing and confirmation of USA300 genotype were performed as described elsewhere [10].

Persistent bacteremia was defined as another positive blood culture >48 hours after the index blood culture in the Edinburgh 2 cohort [14] and >96 hours after the index culture in the IDISA cohort. In the SABG-PCS cohort, persistent bacteremia was defined as persistently positive blood cultures for ≥5 days after initiation of appropriate antimicrobial therapy. Recurrent bacteremia was defined as another positive blood culture within 90 days of stopping treatment in the Edinburgh 2 and SABG-PCS cohorts and within 90 days of the index blood culture in the IDISA cohort. In the Edinburgh 2 cohort, this was restricted to bacteremia caused by an isolate from the same *spa*-inferred clonal complex as the index bacteremia.

### Statistical Analysis

LCA was used to identify subgroups within the different cohorts. A detailed description was given elsewhere [6]. Indicator variables used to identify subgroups were the same as in our previous analysis except that endocarditis and other metastatic complications were included as a single variable rather than separately, due to data availability. Classes were formed without consideration of outcomes. Nonnormally distributed variables with right skew were log-transformed. Missing values were handled with full information maximum likelihood. A combination of the bayesian information criteria (BIC), number of classes, size of the smallest class, and clinical interpretation were used for model selection. We first identified models using the BIC (the most conservative index where a decrease indicates a more informative model), then checked smallest class sizes (to avoid uninformative and underpowered classes), and then applied clinical interpretation (eg, to ensure that all classes were clinically distinct).

To avoid a local maximum, 16 random starting values were used, with 50 iterations performed for each starting value [15]. The results were reviewed to confirm that the same maximum likelihood solution was found. Following class identification, we calculated the posterior probability of class membership for each individual across all identified classes, and we assigned each individual to the class with the highest posterior class membership probability. We corrected for misclassification error using bias-adjusted 3-step LCA [16]. LCA was done using the Latent GOLD 6.1 statistical software package [17].

To compare continuous variables, the Mann-Whitney *U* test was used for comparison between 2 cohorts and the Kruskal-Wallis test for comparison between all 3 cohorts. Categorical variables were compared by contingency table analysis, using χ^2^ test if cell counts were ≥5 and Fisher exact test if they were <5. *P* values <.05 was deemed to indicate statistical significance, and *z* scores were used to visualize the comparison of variables and outcomes between subphenotypes, calculated using the following formula: *z* = (value for subphenotype − mean for variable)/SD for variable. GraphPad Prism software ((version 10.5.0 for macOS) was used for data visualization and analysis.

## RESULTS

### Cohort Characteristics

Characteristics of the Edinburgh 2, IDISA, and SABG-PCS cohorts are compared in Table 1. Notable differences between these cohorts included a lower prevalence of chronic kidney disease (CKD) in the Edinburgh 2 cohort and a lower prevalence of people who inject drugs in the IDISA cohort. In the SABG-PCS cohort there was substantially greater multimorbidity (as determined with the CCI), despite a slightly lower median age. Infective endocarditis was diagnosed less frequently in the Edinburgh 2 cohort. MRSA bacteremia was uncommon in the Edinburgh 2 and IDISA cohorts (rates, 2.8% and 2.0%, respectively) compared with the high rate in the SABG-PCS cohort (40.2%). Persistent bacteremia was also more common in the SABG-PCS cohort despite a more stringent definition (requiring positive blood cultures for ≥5 days after starting treatment). Across the cohorts, most episodes of SAB were acquired in the community, and an unknown portal of entry was the most frequent individual category, followed by skin or soft-tissue infection and intravenous catheters.

**Table 1.** Comparison of Cohort Characteristics

Characteristic	Patient, No. (%)^a^			*P* Value^b^
	Edinburgh 2 (n = 463)	IDISA (n = 490)	SABG-PCS (n = 755)	
Location	NHS Lothian, Scotland, United Kingdom	Randstad region, the Netherlands	Duke University Medical Center, North Carolina, United States	…
Time period	Aug 2022 to Dec 2024	Jul 2017 to Sep 2019	Jan 2016 to Jan 2020	…
Age, median (IQR), y^c^	65 (52–79)	68 (57–77)	61 (49–71)	<.001
Male sex^c^	302 (65.2)	327 (66.7)	470 (62.3)	.24
Acquisition^c^				
Community-acquired, non–healthcare associated	199 (43.0)	166 (33.9)	322 (42.6)	<.001
Community-acquired, healthcare associated	120 (25.9)	163 (33.3)	333 (44.1)	
Nosocomial	144 (31.1)	161 (32.9)	100 (13.2)	
Comorbid conditions				
CCI, median (IQR)	4 (2–6)	5 (3–8)	9 (5–11)	<.001
Diabetes mellitus^d^	131 (28.3)	156 (31.8)	293 (38.9)	<.001
Vascular disease^c,e^	121 (26.1)	211 (43.1)	243 (32.2)	<.001
CKD^c^	32 (6.9)	135 (27.6)	145 (19.2)	<.001
Prosthetic cardiac material^c,f^	40 (8.6)	54 (11.0)	102 (13.5)	.03
IDU^c^	49 (10.6)	5 (1.0)	83 (11.1)	<.001
Liver disease^c^	77 (16.6)	26 (5.3)	97 (12.8)	<.001
Dementia^c^	32 (6.9)	19 (3.9)	77 (10.2)	<.001
Vital signs, median (IQR)^g^				
Pulse rate, beats/min^c^	101 (89 to 114)	105 (94–120)	See Supplementary Table 1	<.001
Temperature, °C^c^	38.4 (37.8–39.0)	38.9 (38.3–39.5)	See Supplementary Table 1	<.001
Laboratory values, median (IQR)^g^				
Hemoglobin, g/L^c^	115.0 (98.0–130.0)	112.8 (97.5–128.9)	NA	.90
Creatinine, μmol/L^c^	92.5 (67.5–144.0)	107.0 (76.0–177.5)	See Supplementary Table 1	<.001
CRP, mg/L^c^	140.0 (54.0–240.8)	155.0 (59.0–262.0)	NA	.20
Bacteremia characteristics				
MRSA^c^	13 (2.8)	10 (2.0)	303 (40.2)	<.001
Any metastatic foci^c^	140 (30.2)	210 (42.9)	380 (50.3)	<.001
Infective endocarditis	38 (8.2)	90 (18.4)	172 (22.8)	<.001
Portal of entry^c^				
Unknown	141 (30.5)	158 (32.2)	207 (27.4)	<.001
Intravenous catheter	91 (19.7)	108 (22.0)	74 (9.8)	
SSTI	101 (21.8)	118 (24.1)	132 (17.5)	
IDU	49 (10.6)	5 (1.0)	51 (6.8)	
Other	28 (6.0)	35 (7.1)	236 (31.3)	
Respiratory	23 (5.0)	34 (6.9)	84 (11.1)	
Urinary tract	30 (6.5)	32 (6.5)	22 (2.9)	
Outcomes				
Persistent bacteremia	17 (3.7)	40 (8.2)	207 (27.4)	<.001
Recurrent bacteremia	12 (2.6)	12 (2.5)	29 (3.9)	.32
90-day mortality	114 (24.6)	161 (32.9)	221 (29.3)	.006

Abbreviations: CCI, Charlson Comorbidity Index; CKD, chronic kidney disease; CRP, C-reactive protein; IDISA, Improved Diagnostic Strategies in *Staphylococcus aureus* bacteremia study; IDU, injection drug use; IQR, interquartile range; MRSA, methicillin-resistant *Staphylococcus aureus*; NA, not available; NHS, National Health Service; SABG-PCS, *Staphylococcus aureus* Bacteremia Group Prospective Cohort Study; SSTI, skin or soft-tissue infection.

^a^Data represent no. (%) of patients unless otherwise specified.

^b^ *P* values based on Mann-Whitney *U* test for continuous values (displayed as median [IQR]) compared between 2 cohorts, Kruskal-Wallis test for continuous values compared between 3 cohorts, and χ^2^ test for categorical variables (displayed as no. [%]).

^c^Class-defining variable included in latent class analysis.

^d^Diabetes mellitus of any severity (data available for 754 patients in the SABG-PCS dataset).

^e^Peripheral vascular disease, myocardial infarction, or stroke.

^f^Implanted cardiac devices, including pacemakers and implantable automatic cardioverter-defibrillator and left ventricular assist devices but not including prosthetic heart valves.

^g^Vital signs and laboratory measurements were recorded at the time of the index blood culture (see further details in Methods).

### Identification of Clinical Subphenotypes

**Figure 1.**
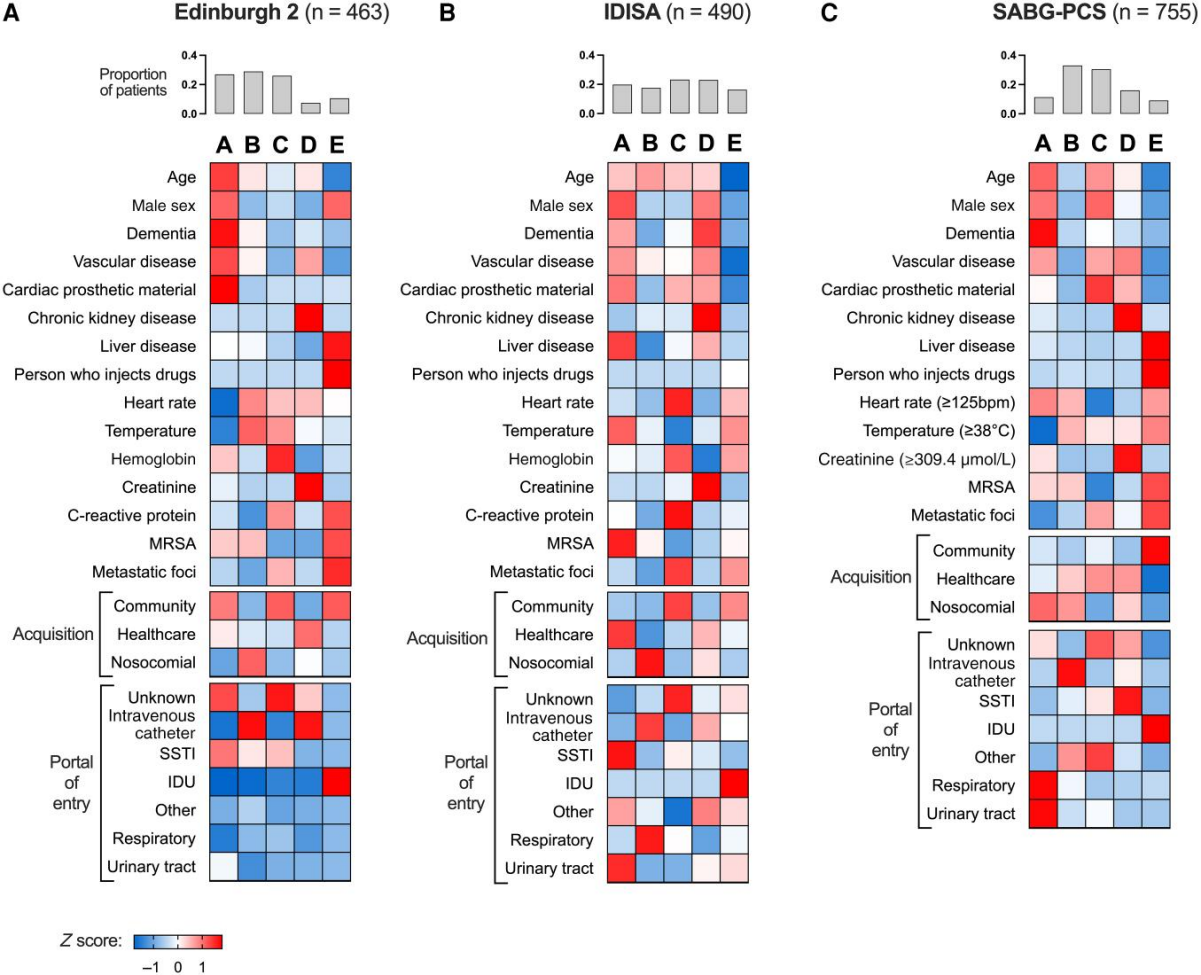
Reproducible clinical subphenotypes of *Staphylococcus aureus* bacteremia. Comparison of class sizes and class-defining variables between subphenotypes for the UK Edinburgh 2 cohort (*A*), the Dutch IDISA (Improved Diagnostic Strategies in *Staphylococcus aureus* bacteremia study) cohort (*B*), and the US SABG-PCS (*Staphylococcus aureus* Bacteremia Group Prospective Cohort Study) cohort (*C*). Letters A–E above columns represent the subphenotypes described in the text. Vertical bar graphs show the proportions of patients assigned to each subphenotype within each cohort. Heat map cells are shaded according to row *z* score (ie, comparing subphenotypes), except for “Acquisition” and “Portal of Entry,” where shading is by column *z* score (ie, comparing within each subphenotype). Intensity of red shading reflects a more positive *z* score (ie, above the mean), and intensity of blue shading reflects a more negative *z* score (ie, below the mean). Abbreviations: IDU, injection drug use; MRSA, methicillin-resistant *S. aureus*; SSTI, skin or soft-tissue infection.

Consistent with our previous work, BIC values, class sizes, and clinical interpretability together favored the 5 class models (Supplementary Table 2). Overall, when class-defining variables were compared between the classes within each cohort, the 5 classes replicated the previously described subphenotypes A–E (Figure 1 and Supplementary Tables 3–5). Subphenotype A was associated with older age, male sex, and multimorbidity (dementia and vascular disease). Subphenotype B was associated with nosocomial SAB, intravenous catheter as the portal of entry, less metastatic infection, and overall less comorbidity. Subphenotype C was associated with community-acquired SAB from an unknown portal of entry ad with metastatic infection and higher C-reactive protein levels. Subphenotype D was associated with CKD, healthcare contact (either community-acquired healthcare-associated or nosocomial acquisition), and vascular disease. Hemodialysis was not included as a class-defining variable but was common in subphenotype D (Edinburgh 2, 16 of 34 cases; IDISA, 22 of 92; SABG-PCS, 76 of 124). Subphenotype E was associated with SAB in younger people without CKD, dementia, or vascular disease. This subphenotype was associated with acquisition through injection drug use (IDU), metastatic infection, and coexistent liver disease.

Certain characteristics were less consistently associated with clinical subphenotype. Of particular clinical relevance, the presence of cardiac prosthetic material was variably associated with subphenotypes A, C, and D across the 3 cohorts. The association of subphenotype A with type of acquisition was also inconsistent (acquisition predominantly community onset and non–healthcare associated in the Edinburgh 2 cohort, community onset and healthcare associated in IDISA, and nosocomial in SABG-PCS).

The contribution of each class-defining variable to the clustering was quantified using the *R*^2^ value (Supplementary Figure 1), indicating that age, acquisition, CKD, IDU, metastatic infection, and portal of entry made the greatest individual contributions to class assignment. MRSA bacteremia was consistently associated with subphenotypes A (old age and multimorbidity), B (nosocomial) and E (IDU) but overall did not make a large contribution to the clustering.

### Comorbidity and Multimorbidity

**Figure 2.**
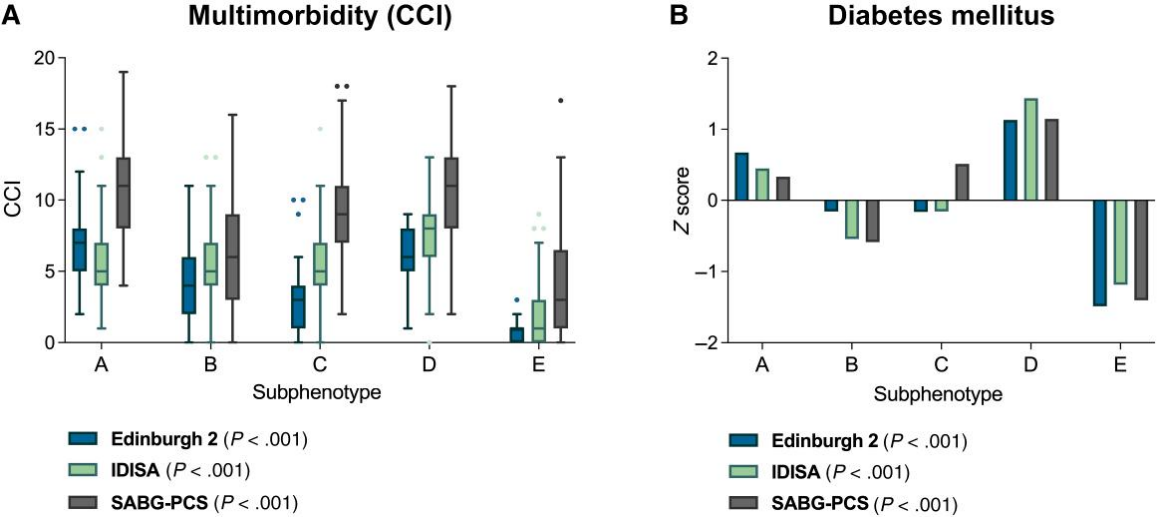
Multimorbidity, measured with the Charlson Comorbidity Index (CCI) (*A*), and diabetes mellitus of any severity (*B*), both stratified by clinical subphenotype. *A*, Box-and-whisker plot drawn using the Tukey method. Boxes shows interquartile range; horizontal lines, median. CCIs were compared between subphenotypes within each cohort, using the Kruskal-Wallis test. *C*, Bars represent *z* scores comparing subphenotypes within each cohort. The raw proportion of patients with diabetes mellitus was compared between subphenotypes within each cohort, using the Fisher exact test. Abbreviations: IDISA, Improved Diagnostic Strategies in *Staphylococcus aureus* bacteremia study; SABG-PCS, *Staphylococcus aureus* Bacteremia Group Prospective Cohort Study.

The extent of multimorbidity, determined using the CCI, differed between the 3 cohorts, with patients in the SABG-PCS cohort having the highest CCI and those in the Edinburgh 2 cohort having the lowest (Table 1 and Supplementary Figure 2). CCIs were compared across subphenotypes, identifying the greatest extent of multimorbidity in subphenotypes A and D, and the lowest in B and E, consistent with our previous findings (Figure 2*A*). Despite the greater baseline multimorbidity, this pattern of differences in CCIs remained in the SABG-PCS cohort. Diabetes mellitus was not included as a class-defining variable but is associated with the risk of acquiring SAB [18]. A diagnosis of diabetes mellitus (of any severity) was consistently associated with subphenotypes D (CKD SAB) and A (older age, multimorbidity SAB) (Figure 2*B*).

### Bacterial Genotype

**Figure 3.**
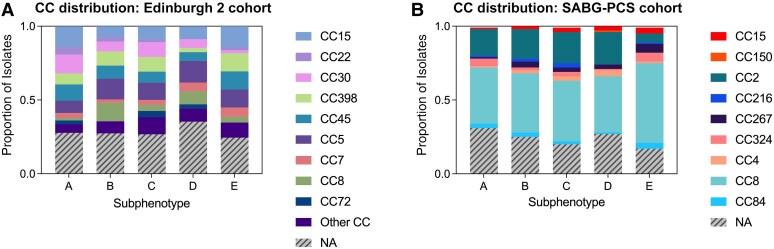
*Staphylococcus aureus spa*-inferred clonal complex (CC) in bloodstream isolates from the Edinburgh 2 cohort (*A*) and the SABG-PCS (*Staphylococcus aureus* Bacteremia Group Prospective Cohort Study) cohort (*B*), stratified by subphenotype. Proportions represent the proportion of isolates within each subphenotype. Abbreviation: NA, not available.

The distribution of *spa*-inferred clonal complexes of isolates from the Edinburgh 2 and SABG-PCS cohorts differed substantially (Supplementary Table 6), with only CC8 and CC15 identified in both cohorts. The distribution of clonal complexes did not differ substantially between subphenotypes within either cohort (Figure 3). Panton-Valentine leukocidin toxin gene carriage was uncommon among isolates from the Edinburgh 2 cohort (7 of 463 isolates, [1.5%]) and did not differ substantially across the subphenotypes (Supplementary Figure 3*A*). The USA300 clone accounted for 14.7% (111 of 755) of isolates in the SABG-PCS cohort. Although numerically more common in subphenotype E, this difference was not statistically significant (*P* = .2) (Supplementary Figure 3*B*).

### Outcomes Associated With Clinical Subphenotypes

**Figure 4.**
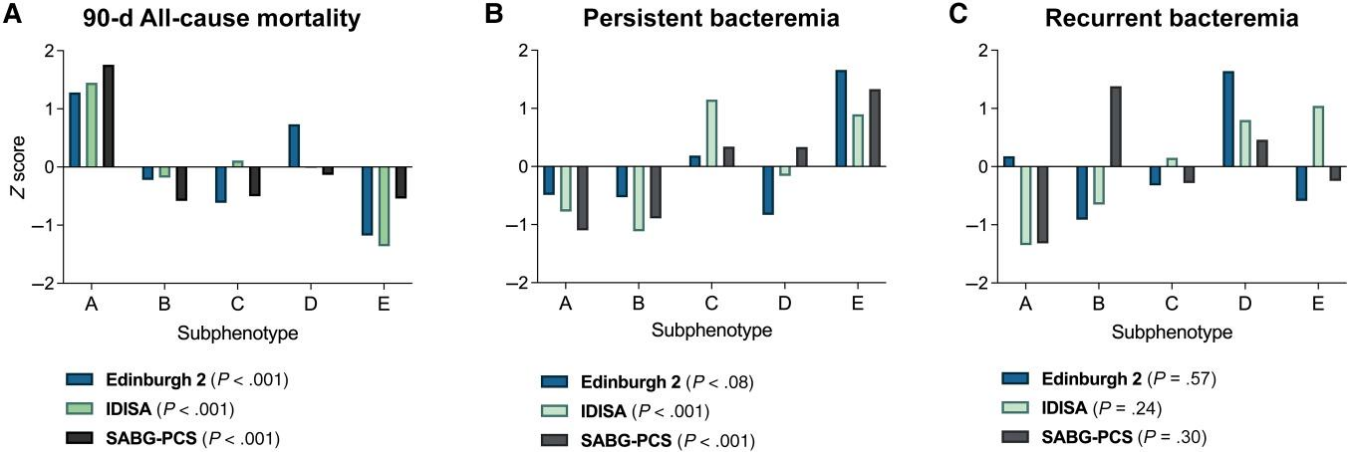
Survival and microbiologic outcomes differ between clinical subphenotypes, as shown by comparison of 90-day all-cause mortality rate (*A*), persistent bacteremia (*B*), and recurrent bacteremia (*C*) by subphenotypes. Bars represent z scores comparing subphenotypes within each cohort. The raw proportion of each outcome was compared between subphenotypes within each cohort using Fisher exact test. Abbreviations: IDISA, Improved Diagnostic Strategies in *Staphylococcus aureus* bacteremia study; SABG-PCS, *Staphylococcus aureus* Bacteremia Group Prospective Cohort Study.

Subphenotype A was consistently associated with highest 90-day all-cause mortality rate (47.9% for Edinburgh 2, 78.3% for IDISA, and 77.9% for SABG-PCS), whereas B (21.1%, 39.4%, and 20.2%, respectively) and especially E (4.0%, 11.2%, and 21.2%) were associated with the lowest (Figure 4*A,*  Supplementary Figure 4, and Supplementary Tables 3–5). Persistent bacteremia was most common in subphenotypes C (4.1% for Edinburgh 2, 16.1% for IDISA, and 36.0% for SABG-PCS) and E (10.2%, 14.5%, and 53.7%, respectively) (Figure 4*B* and Supplementary Tables 3–5). Subphenotype D was consistently associated with recurrent bacteremia, although this difference was not statistically significant (Figure 4*C* and Supplementary Tables 3–5).

## DISCUSSION

Approaches to achieve patient stratification remain an unmet need in SAB, to facilitate personalized clinical management and to address the heterogeneity in responses to treatments investigated in clinical trials. In the current study we have analyzed 3 cohorts of adults with SAB, including a total of 1708 patients, replicating the 5 clinical subphenotypes we described elsewhere [6]. Importantly, we included geographically diverse observational studies reflecting “real-world” patients with SAB, including varied *S. aureus* genetic lineages, and a high proportion of MRSA and USA300 bacteremia. Consistent with our original report, subphenotypes represent 5 clinically distinct archetypes of SAB [7], and they differ in survival and microbiologic outcomes. The reproducible identification of clinically relevant subphenotypes supports the contention that investigation of SAB as a single syndrome is overly simplistic and may be impeding efforts to personalize care and improve outcomes.

Multimorbidity, and diabetes mellitus specifically, was more prevalent in the subphenotypes associated with highest mortality rates (A and D), highlighting the need to understand how these processes affect host defense and response to treatment. Immune dysfunction associated with CKD could also be a specific contributor to the increased mortality risk and potentially to recurrence in subphenotype D. This would be consistent with the overall increased risk of infection-related death in people with CKD, including in comparison with other comorbid conditions [19–21]. Identification of metastatic foci was more prevalent in subphenotypes C (community acquired metastatic SAB) and E (IDU-associated SAB), which were consistently associated with persistent bacteremia but not with death. The predisposition to metastatic infection in subphenotype C remains unclear, not related to the host or pathogen characteristics investigated in this study. Investigation of host responses and the impact of pathogen genetic variation at the whole-genome level in this subphenotype could be particularly informative to define host and/or pathogen determinants of metastatic infection during SAB.

Our study has several strengths, providing confidence in our conclusions. The current analysis includes a large cohort from the United States with a high prevalence of MRSA bacteremia (and the USA300 clone), which was missing from our original study. Compared with other variables, MRSA infection itself did not have a large impact on class assignment, providing reassurance that the subphenotypes are generalizable to settings with a higher prevalence of MRSA. Related to this, the distribution of *S. aureus* genetic lineages was almost entirely different in the Edinburgh 2 versus SABG-PCS cohorts, providing further reassurance regarding generalizability. We studied 3 well-characterized patient cohorts which used the same definitions of the class-defining variables used in the analysis. There was a very high degree of overlap in availability of the variables, and all patients had follow-up extending ≥90 days after the date of the index blood culture. Two of the 3 cohort studies were prospective. The use of 3 observational cohorts provides confidence that these subphenotypes are applicable to patients encountered during “real-life” clinical practice, which can differ from patients included in clinical trials [5, 22].

Subjectivity in model selection represents an important limitation to our approach. For the Edinburgh 2 cohort, the 5-class model had the lowest BIC value and best clinical interpretability. In both the IDISA and SABG-PCS cohorts, although the BIC value marginally favored the 4-class models, clinical interpretability favored the 5-class models, with an acceptable number of patients in the smallest class and no significant differences in entropy (as a measure of class separation). In addition, the study time periods were not overlapping between cohorts, data collection was retrospective in the Edinburgh 2 cohort, and definitions of persistent SAB differed. Despite the differences in the definition of persistent SAB, consistent associations with subphenotypes were observed, supporting the robustness of these associations.

The competing risk of death was not considered when examining associations between subphenotype membership and persistent or recurrent SAB. The low number of recurrences in each cohort prevented statistical confirmation of a difference between the subphenotypes. Granular definitions of sepsis (such as the Sequential Organ Failure Assessment score) were not recorded for all cohorts so could not be included in the model nor compared between subphenotypes. Future work will define a prediction model for use in clinical practice and research studies for subphenotype assignment of individual patients. This will then allow prospective evaluation of the impact of subphenotype assignment on personalizing diagnostic and therapeutic strategies in clinical trials.

An alternative approach has recently been reported by Gutiérrez-Gutiérrez and colleagues [23], with a focus on early clinical risk stratification using variables available within the first 24 hours after the index blood culture. This approach identified 3 clusters based on portal of entry (skin or soft tissue; intravascular, mainly catheter related; and other/unknown) with subsequent identification of clusters within each with higher or lower 30-day mortality risk. We agree that this is a useful and carefully validated tool for clinicians seeking to stratify the risk of death early in infection. This differs from and complements our approach, which has defined holistic subphenotypes of disease which differ in mortality risk and also microbiologic outcomes and response to adjunctive rifampicin (in the ARREST trial).

In summary, we report 5 reproducible clinical subphenotypes of SAB that provide a framework to rationalize the substantial heterogeneity intrinsic to this high morbidity and mortality infection. The current study extends the total number of patients included in our analyses to 3138, spanning 5 separate studies from the United Kingdom, Spain, the Netherlands, and the United States. We have also established the relevance of these subphenotypes in the setting of MRSA bacteremia, the USA300 clone, and patients with a spectrum of multimorbidity. We think that these subphenotypes are robust and can be used to stratify recruitment to and analysis of clinical trials in SAB, in addition to investigation of disease pathogenesis.

## Supplementary Material

ciaf655_Supplementary_Data

## Supplementary Data

Supplementary materials are available at *Clinical Infectious Diseases* online. Consisting of data provided by the authors to benefit the reader, the posted materials are not copyedited and are the sole responsibility of the authors, so questions or comments should be addressed to the corresponding author.

## References

[1] Tong SYC, Fowler VG Jr, Skalla L, Holland TL. Management of *Staphylococcus aureus* bacteremia: a review. JAMA 2025; 334:798–808. 40193249 10.1001/jama.2025.4288 PMC12663922

[2] GBD 2019 Antimicrobial Resistance Collaborators . Global mortality associated with 33 bacterial pathogens in 2019: a systematic analysis for the Global Burden of Disease Study 2019. Lancet 2022; 400:2221–48. 36423648 10.1016/S0140-6736(22)02185-7 PMC9763654

[3] Russell CD, Berry K, Cooper G, et al Distinct clinical endpoints of *Staphylococcus aureus* bacteraemia complicate assessment of outcome. Clin Infect Dis 2024; 79:604–11. 38767234 10.1093/cid/ciae281 PMC11426269

[4] Grillo S, Pujol M, Miró JM, et al Cloxacillin plus fosfomycin versus cloxacillin alone for methicillin-susceptible *Staphylococcus aureus* bacteremia: a randomized trial. Nat Med 2023; 29:2518–25. 37783969 10.1038/s41591-023-02569-0 PMC10579052

[5] Dolby HW, Clifford SA, Laurenson IF, Fowler VG, Russell CD. Heterogeneity in *Staphylococcus aureus* bacteraemia clinical trials complicates interpretation of findings. J Infect Dis 2022; 226:723–8. 35639909 10.1093/infdis/jiac219 PMC9441204

[6] Swets MC, Bakk Z, Westgeest AC, et al Clinical sub-phenotypes of *Staphylococcus aureus* bacteraemia. Clin Infect Dis 2024; 79:1153–61. 38916975 10.1093/cid/ciae338 PMC11581694

[7] Baillie JK, Angus D, Burnham K, et al Causal inference can lead us to modifiable mechanisms and informative archetypes in sepsis. Intensive Care Med 2024; 50:2031–42. 39432104 10.1007/s00134-024-07665-4 PMC7616750

[8] Thwaites GE, Scarborough M, Szubert A, et al Adjunctive rifampicin for *Staphylococcus aureus* bacteraemia (ARREST): a multicentre, randomised, double-blind, placebo-controlled trial. Lancet 2018; 391:668–78. 29249276 10.1016/S0140-6736(17)32456-X PMC5820409

[9] Bai AD, Lo CKL, Komorowski AS, et al *Staphylococcus aureus* bacteraemia mortality: a systematic review and meta-analysis. Clin Microbiol Infect 2022; 28:1076–84. 35339678 10.1016/j.cmi.2022.03.015

[10] Souli M, Ruffin F, Choi S-H, et al Changing characteristics of *Staphylococcus aureus* bacteremia: results from a 21-year, prospective, longitudinal study. Clin Infect Dis 2019; 69:1868–77. 31001618 10.1093/cid/ciz112 PMC6853684

[11] Kempker RR, Farley MM, Ladson JL, Satola S, Ray SM. Association of methicillin-resistant *Staphylococcus aureus* (MRSA) USA300 genotype with mortality in MRSA bacteremia. J Infect 2010; 61:372–81. 20868707 10.1016/j.jinf.2010.09.021 PMC2975870

[12] van der Vaart TW, Prins JM, Soetekouw R, et al All-cause and infection-related mortality in *Staphylococcus aureus* bacteremia, a multicenter prospective cohort study. Open Forum Infect Dis 2022; 9:ofac653. 36589483 10.1093/ofid/ofac653 PMC9792080

[13] Friedman ND, Kaye KS, Stout JE, et al Health care–associated bloodstream infections in adults: a reason to change the accepted definition of community-acquired infections. Ann Intern Med 2002; 137:791–7. 12435215 10.7326/0003-4819-137-10-200211190-00007

[14] Kuehl R, Morata L, Boeing C, et al Defining persistent *Staphylococcus aureus* bacteraemia: secondary analysis of a prospective cohort study. Lancet Infect Dis 2020; 20:1409–17. 32763194 10.1016/S1473-3099(20)30447-3

[15] Magdison JKVJ . Technical guide for latent GOLD 5.1: basic, advanced, and syntax. Belmont, MA: Statistical Innovations, 2016.

[16] Vermunt JK . Latent class modeling with covariates: two improved three-step approaches. Polit Anal 2010; 18:450–69.

[17] Vermunt JK, Magidson J. LG-syntax user's guide: manual for latent gold syntax module version 6.0. Arlington, MA: Statistical Innovations, 2021.

[18] Laupland KB, Pasquill K, Dagasso G, Parfitt EC, Steele L, Schonheyder HC. Population-based risk factors for community-onset bloodstream infections. Eur J Clin Microbiol Infect Dis 2020; 39:753–8. 31858354 10.1007/s10096-019-03777-8

[19] Syed-Ahmed M, Narayanan M. Immune dysfunction and risk of infection in chronic kidney disease. Adv Chronic Kidney Dis 2019; 26:8–15. 30876622 10.1053/j.ackd.2019.01.004

[20] Wang HE, Gamboa C, Warnock DG, Muntner P. Chronic kidney disease and risk of death from infection. Am J Nephrol 2011; 34:330–6. 21860228 10.1159/000330673 PMC3169360

[21] Mansur A, Mulwande E, Steinau M, et al Chronic kidney disease is associated with a higher 90-day mortality than other chronic medical conditions in patients with sepsis. Sci Rep 2015; 5:10539. 25995131 10.1038/srep10539 PMC4650757

[22] Cooper G, Dolby HW, Berry K, Russell CD. Eligibility of patients with *Staphylococcus aureus* bacteraemia for early oral switch. Lancet Infect Dis 2024; 24:e209–10. 38309279 10.1016/S1473-3099(24)00065-3

[23] Gutiérrez-Gutiérrez B, Gallego-Mesa B, Kaasch AJ, et al Identification and validation of clinical phenotypes in *Staphylococcus aureus* bloodstream infection and their association with mortality (FEN-AUREUS study). eClinicalMedicine 2025; 83:103240. 40630616 10.1016/j.eclinm.2025.103240 PMC12235390

