# Supplementary material for: Reproducible Identification of Staphylococcus aureus Bacteremia Clinical Subphenotypes
Source: Clin Infect Dis. 2025 Nov 27;82(4):e643–51. doi: 10.1093/cid/ciaf655 (PMC13131937; doi:10.1093/cid/ciaf655)
Supplement: ciaf655_Supplementary_Data [file ciaf655_supplementary_data.pdf]

Supplementary material

Reproducible identification of *Staphylococcus aureus* bacteremia clinical subphenotypes

Contents:

Supplementary Table 1: Ordinal variables in SABG-PCS dataset 2
Supplementary Table 2: Model fit statistics 3
Supplementary Table 3: Edinburgh 2 cohort subphenotypes raw data 4
Supplementary Table 4: IDISA cohort subphenotypes raw data 5
Supplementary Table 5: SABG-PCS cohort subphenotypes raw data 6
Supplementary Table 6: *S. aureus* clonal complex distributions in Edinburgh and SABG-PCS cohorts 7
Supplementary Figure 1: Contribution of class-defining variables to clustering 8
Supplementary Figure 2: Multimorbidity in the SABG-PCS, Edinburgh, and IDISA cohorts. 9
Supplementary Figure 3: Additional bacterial characteristics 10
Supplementary Figure 4: Survival curve of subphenotypes in Edinburgh 2 cohort 11

Supplementary Table 1: Ordinal variables in SABG-PCS dataset

Variable	Category	N (%)
Heart rate, beats/min		
	≤39	10 (1.3)
	40-54	33 (4.4)
	55-69	64 (8.5)
	70-109	265 (35.1)
	110-139	261 (34.6)
	140-179	112 (14.8)
	≥180	10 (1.3)
Temperature, °C		
	≤29.9	<5
	30-31.9	<5
	32-33.9	15 (2.0)
	34-35.9	33 (4.4)
	36-38.4	358 (47.5)
	38.5-38.9	120 (15.9)
	39-40.9	216 (28.6)
	≥41	7 (0.9)
Creatinine, μM		
	<53	36 (4.8)
	53-128	354 (46.9)
	129-180	113 (15.0)
	181- 305	119 (15.8)
	>305	133 (17.6)

SABG-PCS: *Staphylococcus aureus* Bacteremia Group - Prospective Cohort Study

Supplementary Table 2: Model fit statistics

Model	BIC	LL	Npar	Entropy	Patients per class						
**Edinburgh 2 cohort**											
					**1**	**2**	**3**	**4**	**5**	**6**	**7**
1-class	14761.6	-7291.8	29	1	463	-	-	-	-	-	-
2-class	14269.7	-6953.7	59	0.96	409	54	-	-	-	-	-
3-class	14058.6	-6756.1	89	0.85	209	173	81	-	-	-	-
4-class	13961.6	-6615.6	119	0.83	162	125	127	49	-	-	-
5-class	13919.8	-6502.6	149	0.86	137	123	120	49	34	-	-
6-class	13963.5	-6432.4	179	0.86	131	107	85	56	51	33	-
7-class	14034.2	-6375.7	209	0.85	102	93	70	77	49	38	34
**IDISA cohort**											
					**1**	**2**	**3**	**4**	**5**	**6**	**7**
1-class	12960.7	-6393.2	29	1.0	490	-	-	-	-	-	-
2-class	12774.0	-6209.7	59	0.65	308	182	-	-	-	-	-
3-class	12650.2	-6057.7	89	0.81	208	158	124	-	-	-	-
4-class	12634.3	-5959.6	119	0.81	160	141	115	74	-	-	-
5-class	12708.6	-5906.6	149	0.81	114	113	97	86	80	-	-
6-class	12805.1	-5864.7	179	0.84	160	121	62	50	54	43	-
7-class	12899.0	-5821.6	209	0.84	109	93	77	76	55	54	26
**SABG-PCS cohort**											
					**1**	**2**	**3**	**4**	**5**	**6**	**7**
1-class	18080.2	-8920.8	36	1	755	-	-	-	-	-	-
2-class	17606.1	-8561.1	73	0.81	641	114	-	-	-	-	-
3-class	17474.8	-8372.9	110	0.83	445	240	70	-	-	-	-
4-class	17440.0	-8232.9	147	0.80	287	274	128	66	-	-	-
5-class	17478.3	-8129.5	184	0.81	244	238	124	80	69	-	-
6-class	17566.5	-8051.0	221	0.81	158	186	147	123	73	68	-
7-class	17696.2	-7993.2	258	0.81	192	150	118	98	71	68	58

Model fit statistics and class sizes for latent class models from one to seven classes. BIC: Bayesian Information Criteria, defined in Methods. LL: Log-likelihood, measures the fit of the model to the data. Npar: number of parameters, measure of model complexity. Entropy is a measure for class separation: it ranges from zero to one and values of ≥0·8 indicate good separation of the different classes.

IDISA: Improved Diagnostic Strategies in *Staphylococcus aureus* bacteremia study; SABG-PCS: *Staphylococcus aureus* Bacteremia Group - Prospective Cohort Study.

Supplementary Table 3: Edinburgh 2 cohort subphenotypes raw data

	Subphenotype				
	**A**	**B**	**C**	**D**	**E**
Characteristics					
Age, years	76.6	62.4	57.3	62.3	43.0
Sex, male	0.80	0.55	0.59	0.53	0.80
Dementia	0.18	0.06	0.00	0.03	0.00
Vascular disease	0.52	0.26	0.06	0.39	0.00
Cardiac prosthetic material	0.25	0.01	0.03	0.03	0.04
Chronic kidney disease	0.02	0.00	0.00	0.87	0.00
Liver disease	0.17	0.17	0.10	0.03	0.41
Person who injects drugs	0.00	0.00	0.00	0.00	1.00
Heart rate, beats/min	94.81	104.96	103.36	103.45	101.70
Temperature, °C	36.3	36.5	36.5	36.4	36.4
Haemoglobin, g/L	115.2	107.5	126.1	100.4	107.5
Creatinine, μM	137.1	77.9	87.3	452.8	70.5
C-reactive protein, mg/L	102.7	68.1	144.9	101.9	161.8
MRSA	0.04	0.04	0.00	0.00	0.06
Metastatic foci	0.22	0.08	0.48	0.23	0.73
Acquisition					
Community-acquired nonhealthcare-associated	0.42	0.00	0.82	0.04	0.94
Community-acquired healthcare-associated	0.35	0.23	0.17	0.64	0.06
Nosocomial	0.24	0.77	0.00	0.32	0.00
Portal of entry					
Unknown	0.41	0.09	0.57	0.24	0.00
Intravenous catheter	0.01	0.52	0.00	0.56	0.00
Skin or soft tissue infection	0.35	0.18	0.26	0.06	0.00
Injection drug use	0.00	0.00	0.00	0.00	1.00
Other	0.07	0.10	0.04	0.06	0.00
Respiratory	0.02	0.08	0.07	0.03	0.00
Urinary tract	0.14	0.03	0.05	0.06	0.00
Outcomes					
Persistent bacteremia	0.04	0.01	0.04	0	0.10
Recurrent bacteremia	0.03	0.02	0.03	0.06	0.02
90-day mortality	0.48	0.21	0.14	0.38	0.04

Data shown as proportion within subphenotype unless otherwise stated. Age, heart rate, temperature, and lab results are shown as mean for each subphenotype.

Supplementary Table 4: IDISA cohort subphenotypes raw data

	Subphenotype				
	A	B	C	D	E
Characteristics					
Age, years	68.3	71.6	68.0	67.0	42.1
Sex, male	0.78	0.61	0.61	0.75	0.56
Dementia	0.06	0.00	0.03	0.09	0.00
Vascular disease	0.55	0.42	0.40	0.57	0.06
Cardiac prosthetic material	0.17	0.05	0.14	0.15	0.00
Chronic kidney disease	0.00	0.17	0.15	0.93	0.02
Liver disease	0.10	0.00	0.05	0.07	0.03
Person who injects drugs	0.00	0.00	0.00	0.00	0.08
Heart rate, beats/min	107.7	106.3	115.1	105.7	110.9
Temperature, °C	39.2	38.8	38.4	38.8	39.1
Haemoglobin, g/L	114.1	112.8	124.1	102.1	119.7
Creatinine, μM	96.3	91.6	122.5	305.7	69.1
C-reactive protein, mg/L	132.2	49.5	274.2	86.1	119.0
MRSA	0.05	0.02	0.00	0.01	0.02
Metastatic foci	0.31	0.13	0.82	0.28	0.64
Acquisition					
Community-acquired nonhealthcare-associated	0.21	0.16	0.71	0.18	0.58
Community-acquired healthcare-associated	0.67	0.05	0.23	0.47	0.31
Nosocomial	0.12	0.79	0.06	0.35	0.11
Portal of entry					
Unknown	0.10	0.25	0.65	0.30	0.38
Intravenous catheter	0.03	0.43	0.00	0.29	0.19
Skin or soft tissue infection	0.52	0.11	0.26	0.20	0.11
Injection drug use	0.00	0.00	0.00	0.00	0.08
Other	0.10	0.07	0.02	0.11	0.09
Respiratory	0.05	0.13	0.07	0.03	0.07
Urinary tract	0.18	0.00	0.00	0.08	0.09
Outcomes					
Persistent bacteremia	0.04	0.01	0.16	0.08	0.15
Recurrent bacteremia	0.0	0.01	0.03	0.04	0.05
90-day mortality	0.78	0.39	0.46	0.43	0.11

Data shown as proportion within subphenotype unless otherwise stated. Age, heart rate, temperature and lab results are shown as mean for each subphenotype.
IDISA: Improved Diagnostic Strategies in *Staphylococcus aureus* bacteremia study

Supplementary Table 5: SABG-PCS cohort subphenotypes raw data

	Subphenotype				
	A	B	C	D	E
Characteristics					
Age, years	71.4	49.5	67.0	58.1	37.5
Sex, male	0.72	0.53	0.73	0.61	0.49
Dementia	0.26	0.07	0.11	0.08	0.04
Vascular disease	0.44	0.16	0.43	0.48	0.10
Cardiac prosthetic material	0.12	0.04	0.27	0.17	0.00
Chronic kidney disease	0.15	0.02	0.01	0.99	0.09
Liver disease	0.13	0.08	0.06	0.09	0.61
Person who injects drugs	0.00	0.04	0.02	0.00	1.00
Heart rate ≥125 beats/min	0.65	0.60	0.33	0.46	0.63
Temperature ≥38°C	0.21	0.51	0.46	0.47	0.56
Creatinine >309.4μM	0.29	0.03	0.04	0.71	0.07
MRSA	0.42	0.42	0.37	0.40	0.45
Metastatic foci	0.13	0.35	0.70	0.48	0.90
Acquisition					
Community-acquired nonhealthcare-associated	0.41	0.35	0.44	0.33	0.85
Community-acquired healthcare-associated	0.36	0.45	0.52	0.51	0.12
Nosocomial	0.23	0.20	0.04	0.16	0.03
Portal of entry					
Unknown	0.29	0.19	0.38	0.33	0.14
Intravenous catheter	0.01	0.25	0.00	0.08	0.00
Skin or soft tissue infection	0.04	0.13	0.19	0.43	0.02
Injection drug use	0.00	0.00	0.00	0.00	0.74
Other	0.06	0.29	0.38	0.13	0.04
Respiratory	0.48	0.12	0.02	0.04	0.06
Urinary tract	0.13	0.01	0.03	0.00	0.00
Outcomes					
Persistent bacteremia	0.10	0.14	0.36	0.36	0.54
Recurrent bacteremia	0.01	0.06	0.03	0.04	0.03
90-day mortality	0.78	0.20	0.22	0.31	0.21

Data shown as proportion within subphenotype apart from age. Age is shown as mean for each subphenotype.
SABG-PCS: *Staphylococcus aureus* Bacteremia Group - Prospective Cohort Study

Supplementary Table 6: *S. aureus* clonal complex distributions in Edinburgh and SABG-PCS cohorts

Clonal complex	Edinburgh 2	SABG-PCS
1	19 (4.2)	0
2	0	145 (19.2)
4	0	18 (2.4)
5	54 (11.8)	0
7	16 (3.5)	0
8	29 (6.3)	309 (40.9)
12	5 (1.1)	0
15	48 (10.5)	23 (3.0)
20	1 (0.2)	0
22	13 (2.8)	0
30	39 (8.5)	0
45	42 (9.2)	0
59	6 (1.3)	0
72	9 (2.0)	0
84	0	19 (2.5)
97	3 (0.7)	0
101	3 (0.7)	0
121	1 (0.2)	0
130	2 (0.4)	0
150	0	3 (0.4)
216	0	14 (1.9)
267	0	28 (3.7)
324	0	20 (2.6)
398	41 (9.0)	0
Not available	126 (27.6)	176 (23.3)

Data shown as n(%).
SABG-PCS: *Staphylococcus aureus* Bacteremia Group - Prospective Cohort Study

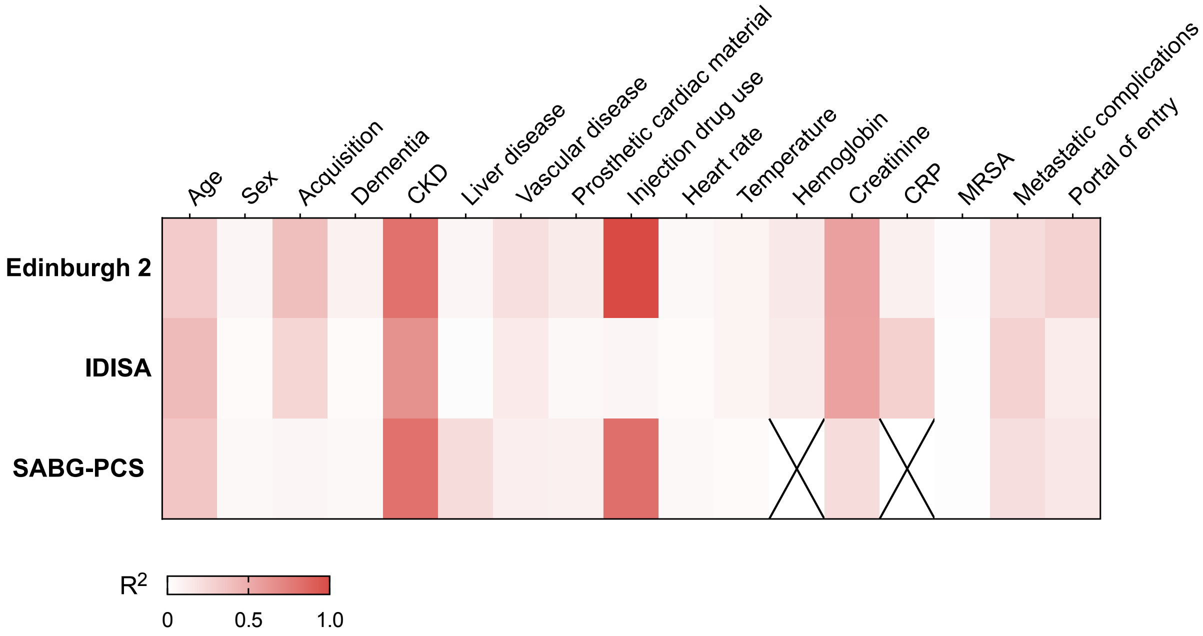

Supplementary Figure 1: Contribution of class-defining variables to clustering

Heatmap representing R^2 values. The R^2 value reflects the contribution to the clustering. Values closer to 1 indicate that the variable plays a stronger role in distinguishing between classes. Values in cells are the R^2 values. Cells marked "X" indicate the variable was not available therefore not used in the analysis.

Abbreviations: IDISA, Improved Diagnostic Strategies in *Staphylococcus aureus* bacteremia study; SABG-PCS, *Staphylococcus aureus* Bacteremia Group - Prospective Cohort Study.

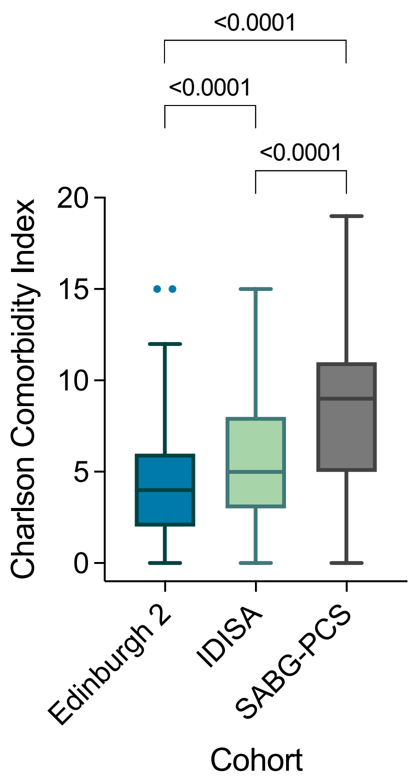

Supplementary Figure 2: Multimorbidity in the SABG-PCS, Edinburgh, and IDISA cohorts.
Box and whisker plot drawn using Tukey's method. Box shows interquartile range and horizontal line shows median. CCI compared between groups using the Kruskal-Wallis test with Dunn's multiple comparisons test. Abbreviations: IDISA, Improved Diagnostic Strategies in *Staphylococcus aureus* bacteremia study; SABG-PCS, *Staphylococcus aureus* Bacteremia Group - Prospective Cohort Study.

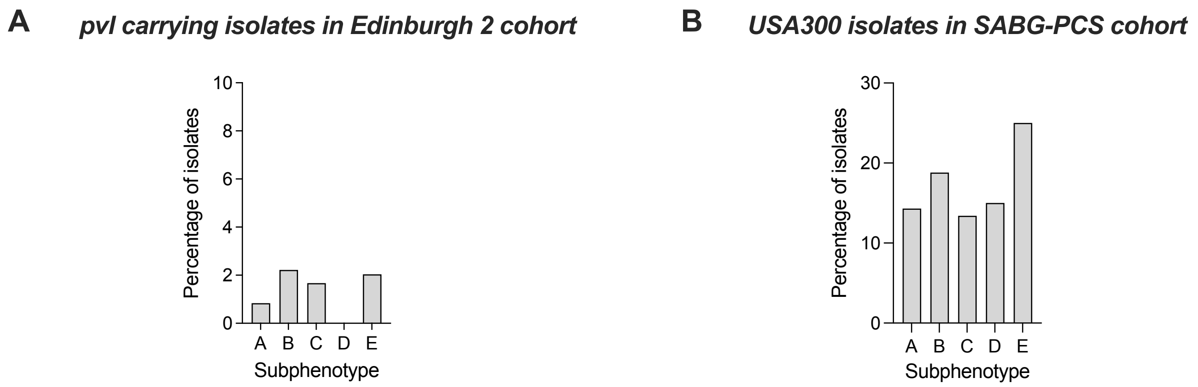

Supplementary Figure 3: Additional bacterial characteristics
(A) Percentage of *S. aureus* bloodstream isolates from Edinburgh 2 cohort carrying the *pvl* toxin genes, stratified by subphenotype. **(B)** Percentage of *S. aureus* bloodstream isolates from the SABG-PCS cohort belonging to the USA300 clone, stratified by subphenotype. Percentage refers to the percentage of isolates within each subphenotype.
Abbreviations: SABG-PCS, *Staphylococcus aureus* Bacteremia Group - Prospective Cohort Study.

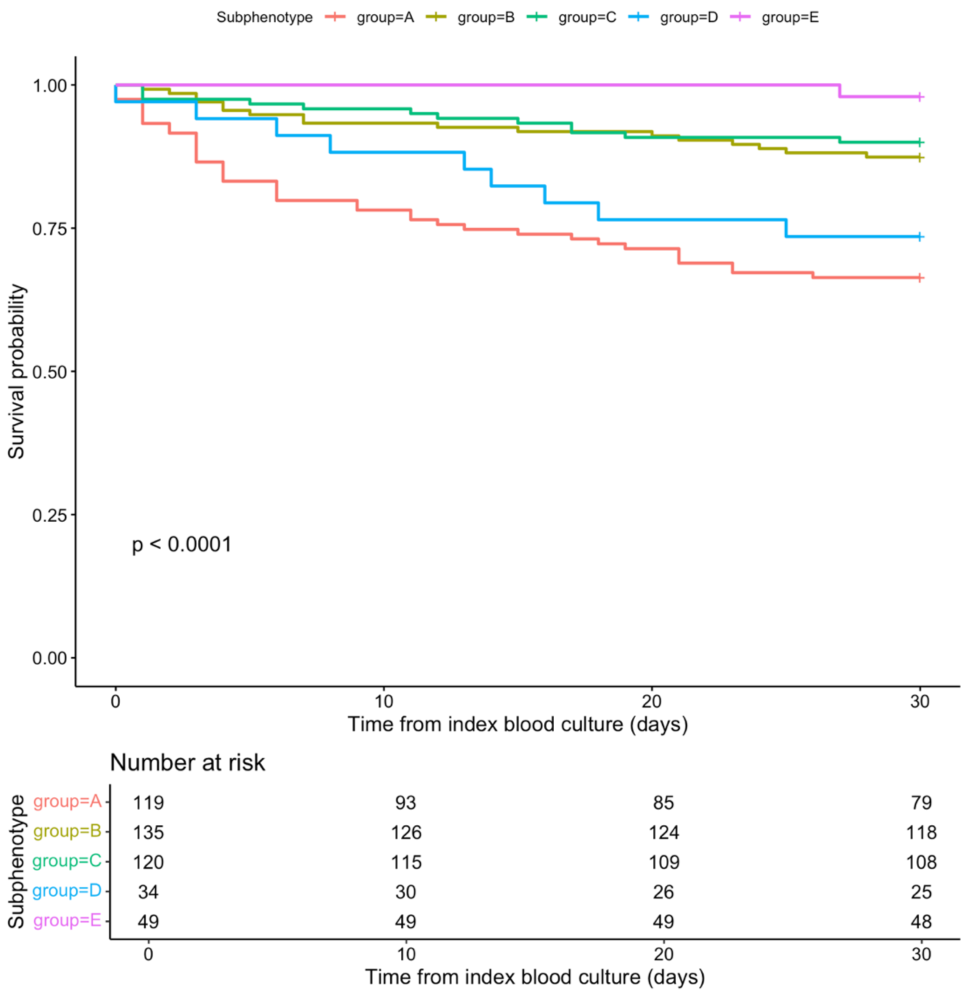

Supplementary Figure 4: Survival curve of subphenotypes in Edinburgh 2 cohort
Kaplan-Meier survival curve for unadjusted 30-day survival. Subphenotypes compared using the log-rank test. Group designations correspond to subphenotypes.
